# The Use of Adipose-Derived Stem Cells in Selected Skin Diseases (Vitiligo, Alopecia, and Nonhealing Wounds)

**DOI:** 10.1155/2017/4740709

**Published:** 2017-08-21

**Authors:** Agnieszka Owczarczyk-Saczonek, Anna Wociór, Waldemar Placek, Wojciech Maksymowicz, Joanna Wojtkiewicz

**Affiliations:** ^1^Department of Dermatology, Sexually Transmitted Diseases and Clinical Immunology, University of Warmia and Mazury in Olsztyn, Olsztyn, Poland; ^2^Foundation for Nerve Cell Regeneration, University of Warmia and Mazury in Olsztyn, Olsztyn, Poland; ^3^Department of Neurology and Neurosurgery, Faculty of Medical Sciences, University of Warmia and Mazury in Olsztyn, Olsztyn, Poland; ^4^Department of Pathophysiology, Faculty of Medical Sciences, University of Warmia and Mazury in Olsztyn, Olsztyn, Poland; ^5^Laboratory for Regenerative Medicine, Faculty of Medical Sciences, University of Warmia and Mazury in Olsztyn, Olsztyn, Poland

## Abstract

The promising results derived from the use of adipose-derived stem cells (ADSCs) in many diseases are a subject of observation in preclinical studies. ADSCs seem to be the ideal cell population for the use in regenerative medicine due to their easy isolation, nonimmunogenic properties, multipotential nature, possibilities for differentiation into various cell lines, and potential for angiogenesis. This article reviews the current data on the use of ADSCs in the treatment of vitiligo, various types of hair loss, and the healing of chronic wounds.

## 1. Introduction

Brown and white adipose tissue is a source of mesenchymal stem cells, specifically adipose-derived stem cells (ADSCs). It is an inexpensive, unlimited reservoir of stem cells. From 300 ml of adipose tissue, 2-3 × 10^8^ ADSCs can be obtained. This is between 100 and 1000 times more than the mesenchymal stem cells from the bone marrow [1–4]. In addition, they can be easily obtained with no ethical dilemmas pertaining to their use [5, 6]. Moreover, the high content of ADSCs in adipose tissue precludes the need for long in vitro culture, which reduces the risk of chromosomal abnormalities [7].

In 2001, researchers at the University of California, Los Angeles described the isolation of a new population of adult stem cells from liposuctioned adipose tissue. These cells were given the name of processed lipoaspirate or PLA cells due to their derivation from processed lipoaspirate tissue obtained through cosmetic surgery. Since then, intensive studies in the use of regenerative medicine have begun. The term PLA cell has now been replaced with the term adipose-derived stem cells or ASCs to give the field some sort of conformity in terms of nomenclature. By and large, the method of ADSC isolation from lipoaspirates using this enzymatic method has not changed significantly [2, 8–10]. Typical isolation procedures for ASCs involve digestion of the lipoaspirated tissue with collagenase and subsequent centrifugation, then a high-density stromal vascular fraction is produced. Subculturing is then performed to detach the ADSCs from the primary adipocytes [8, 9].

## 2. Physiology of ADSCs

ADSCs are heterogeneous, no specific marker for them has been identified, and the location of stem cells in adipose tissue is difficult to determine. However, most of them occur in the perivascular regions. The morphology of ADSCs resemble fibroblasts, consisting of a large endoplasmic reticulum and nuclei [9]. ADSCs do not have a specific marker and the expression of antigens is similar to bone marrow MSCs: CD10, CD13, CD29, CD34, CD44, CD54, CD71, CD49b, CD90, CD105, CD117, and STRO-1. However, they do not express the hematopoietic markers, such as CD14, CD16, CD31, CD45, CD56, CD61, CD62E, CD104, CD106, CD144, the endothelial cell markers CD31, CD144, and von Willebrand factor [9, 11, 12]. Moreover, they are privileged cells with reduced immunogenicity; therefore, there is no expression of HLA-DR [13, 14].

ADSCs may also be a precursor of chondrocytes, osteocytes, muscle cells, neurons, and fibroblasts as well as keratinocytes under proper conditions. However, their most important function is the stimulation of surrounding cells to differentiate into specialized cells under the influence of certain growth factors [15–17]. It has been shown that the ADSCs are even necessary for the activation of epidermal stem cells in the skin [18]. Their exogenous administration mobilizes other stem cells, including the stem cells of the epidermis from the “bulge” region of the hair follicle. This action is based on the production of growth factors, including epidermal growth factor (EGF), fibroblast growth factor (FGF-*β*), hepatocyte growth factor (HGF), transforming growth factor (TGF-*β*), vascular endothelial growth factor (VEGF), keratinocyte growth factor (KGF), granulocyte-macrophage colony-stimulating factor (GM-CSF), stromal factor 1-alpha, and cytokines, such as IL-6, 8, 11, 12, and TGF-*α*. This paracrine secretion of cytokines explains their high concentrations in obese patients [11, 15, 17]. ADSCs also inhibit the production of proinflammatory cytokines, enhance the production of anti-inflammatory IL-10, and stimulate the regulatory T cells [11, 19]. They also stimulate angiogenesis by differentiation in endothelial cells. ADSCs can protect against apoptosis, which offers great opportunities for their use in regenerative medicine [3, 4, 11, 20]. Expression of the receptor for PDGF and CD10 is constant, regardless of the number of passages [15, 21]. Traktuev et al. showed that cells with the CD34+/CD31 phenotype have the ability to stabilize the endothelial network in vitro and stabilize neovascularization in vivo [15, 22]. In addition, perivascular ADSCs (CD146+) also function as a niche for hematopoietic stem cells in vitro [22].

Of particular interest is platelet-derived growth factor-D (PDGF-D), which is secreted by the ADSCs. It is a mitogen for mesenchymal cells, which induces the transformation of cells and also accelerates tumor growth, but its role is not quite understood. Kim et al. showed that PDGF-D and PDGF receptor *β* are expressed in ADSCs, but PDGF-B is not. PDGF-D can increase the proliferation and migration of ADSCs for the generation of mitochondrial reactive oxygen species (MTRose) and by controlling mRNA expression of various growth factors (VEGF, FGF-1, FGF5, EGF, leukemia inhibitory factor, inhibin, and IL-11) [15, 23].

Cultured ADSCs from this niche have ultrastructural features similar to primitive MSCs (large nucleus, immature cytoplasmic organelles). Although Rubio et al. reported that human ADSCs can undergo malignant transformation during long passages of more than four months, five years later, the authors were not able to reproduce the phenomenon of transformation, most likely due to contamination artifacts [24].

ADSCs have an antioxidant effect. They can capture free radicals and heat shock protein in ischemia status. Research has revealed that during the aging processes and in diabetes, the function of ADSCs is impaired [2, 25].

Vitamins can affect the proliferation of ADSCs. The addition of folic acid and vitamin B12 slightly increases their activity in cell culture, while vitamin C significantly stimulates ADSCs in a dose-dependent manner. Vitamin C increases the expression of the mRNA of HGF, VEGF, bFGF, and KGF [15].

There are some differences in the physiological and biological features of ADSCs derived from different anatomical sites. Siciliano et al. compared the characteristics of stem cells from mediastinal fat and skin. Subcutaneous ADSCs demonstrated greater proliferation and differentiation capacity, an increased IL-6 secretion, and a smaller VEGF-C than ADSCs isolated from the mediastinum. ADSCs from the mediastinum showed a higher proangiogenic potential [26]. On the other hand, ADSCs from the visceral fat have a reduced susceptibility to apoptosis, and ADSCs from the pericardium, omentum, and groin have a different phenotype [27].

Obesity has an influence on the differentiation potential and immunogenicity of ADSCs. The study by Perez et al. demonstrated that stem cells derived from murine and human nonobese sources had increased sensitivity to insulin and can inhibit lipolysis during differentiation into mature adipocytes. In contrast, cells isolated from obese patients showed an impaired uptake of glucose, insulin resistance, and less antilipolytic effect of insulin. Moreover, they released a greater amount of proinflammatory cytokines (mainly TNF-*α*) and showed disturbances in the production of adiponectin [28].

Interestingly, the preferred factor for proliferation, migration, and differentiation of the ADSCs is hypoxia (an oxygen concentration of 1–5%). Hypoxia induces the expression of HIF-1*α* (hypoxia-inducible factor 1-*α*) and increases the production of growth factors, particularly VEGF, bFGF, and HGF which are involved in neovascularization [15, 16]. This phenomenon is observed in obesity. Local hypoxia in the adipose tissue induces the formation of free radicals (ROS) and leads to the secretion of growth factors which stimulate the formation of new blood vessels [15].

Pachon-Pena et al. have also found that obese-derived hADSCs demonstrate increased proliferation and migration capacity, but decreased lipid droplet accumulation, which is correlated with a higher expression of human leukocyte antigen- (HLA-) II, a cluster of CD106 differentiation, and a lower expression of CD29. Of interest, adipogenic differentiation modified CD106, CD49b, and HLA-ABC surface protein expression, which was dependent on the donor's BMI. Moreover, low oxygen tension increased proliferation and migration of lean but not obese hASCs, which was correlated with an altered CD36 and CD49b immunophenotypic profile [29]. Moreover, in obesity, ADSCs indicate changes in their transcriptomic profile (set of mRNA molecules present in a particular point of a cell) with a loss of plasticity, simultaneously showing an increasing similarity to the adipocyte phenotype [15, 30].

Currently, ADSCs are used in aesthetic dermatology for skin rejuvenation, to correct wrinkles, to correct facial lipoatrophy, and even to improve erections. They are described in the treatment of perianal fistulas in Crohn's disease, bone grafts, and type 1 diabetes [20, 31]. However, the therapeutic use of ADSCs is still experimental.

## 3. Vitiligo

Vitiligo is a disorder caused by the loss of melanocytes. Repigmentation of vitiligo depends on available melanocytes from three possible sources: from the hair follicle unit which is the main provider of pigment cells, from the border of vitiligo lesions, and from unaffected melanocytes within depigmented areas [32]. Melanocytes rarely undergo mitosis without growth factors; therefore, mitogenic factors are used in transplantation treatments for this disease [33, 34]. Repigmentation occurs due to the migration of melanocyte stem cells (MelSCs) located in the lower part of the hair follicle (infundibulum). Therefore, this process starts perifolliculary [33–36].

ADSCs can be a source of growth factors for melanocytes cultured in the presence of keratinocytes. Lim et al. showed efficacy in mice and Sprague-Dawley rats after administration of human melanocytes alone or enriched with human ADSCs. Better results have been shown with a coadministration of melanocytes and ADSCs, which were grown separately and then mixed in a ratio of 1 : 1, 1 : 2, or 1 : 3, as compared to the administration of pure melanocytes alone [33].

Although the interaction between ADSCs and melanocytes are well known, in the study of Kim et al., an increase in the secretion of HGF by ADSCs after prior exposure to bFGF or EGF was demonstrated [37]. They showed that the proliferation and migration of melanocytes were significantly stimulated by coculturing the ADSCs in comparison with monoculture melanocytes. This may be related to the presence of bFGF and melanocyte growth factor (MGF) produced by ADSCs [37]. The ratio of melanocytes with positive expression of TRP-2, E-cadherin, and N-cadherin were significantly increased in the cocultures with ADSCs compared to keratinocyte and melanocyte monocultures. Melanocytes with a positive expression of TRP-2 (tautomerase dopachrome) are considered to be melanocyte precursors, but TRP-1 positive is considered to be diverse and mature [37, 38]. This is an important result, because the greater the number of immature melanocytes, the better clinical outcomes. In addition, cadherin-calcium-dependent cell adhesion receptors take part in cell-cell interactions. E-cadherin determines the adhesion between keratinocytes and melanocytes, and N-cadherin facilitates the contact between fibroblasts and melanocytes. They also play a role in the differentiation of melanocytes [37, 39]. These studies have confirmed that cultures with ADSCs increase the proliferation and migration of melanocytes, while reducing their differentiation [37].

## 4. Alopecia

Multipotent stem cells can regenerate hair follicles and sebaceous glands in the skin. The stem cells can be used to regenerate hair growth in a number of therapeutic methods:

- the reversal of pathological mechanisms that contributes to hair loss (androgenetic alopecia);
- complete regeneration of hair follicles with “bulge”;
- neogenesis of hair follicles with a stem cell culture [40, 41].

The newest therapeutic option is the use of ADSCs. Fukuoka and Suga used them in 22 patients (11 men and 11 women) with alopecia. The cells were administered intradermally every 3 to 5 weeks (6 sessions), controlling the growth of hair using a trichogram. They observed significant improvement in both female and male patients [42].

Hair follicles are surrounded by subcutaneous fat cells and skin, which make up the interfollicular dermal macroenvironment, important in maintaining normal cell growth in the region bulges and hair follicles [18, 43]. Moreover, ADSCs are necessary for the activation of epidermal stem cells [18]. Their action is based primarily on the secretion of growth factors, such as VEGF which regulates hair growth and the size of the hair follicle by stimulating angiogenesis, HGF which is engaged in the length of the phases of the hair cycle, PDGF which induces and maintains the anagen phase, and IGF-1 which controls the cycle of hair growth and hair cell differentiation [42, 44–47]. ADSCs stimulate angiogenesis and enhance blood supply to the hair papilla cells. They also have immunomodulatory and immunosuppressive effects through direct interactions between the cells and secrete prostaglandin E2 (PGE2), leukemia inhibitory factor (LIF), and kynurenine [18]. Huang et al. studied the effect of ADSCs on papilla cells of the hair. During the cell culture, the hair retained its own markers. After adding ADSCs (isolated from rats), characteristics common to coculture were observed. There were mixed papilla and medulla cells with ADSCs. The core and the inner shell of the outer coat also contained ADSCs. The best results were achieved in the second cocultures [44].

It was also shown that subcutaneous adipose tissue played an important role in the extension of the anagen phase. There was a proliferation of progenitor cells, which were adipocytes in the transition from the telogen phase to the anagen phase of the hair follicle [43, 44]. The layer thickness of subcutaneous adipocytes during active hair growth (anagen) increased significantly compared to their amount in the resting phase (telogen) [18, 43]. ADSCs stimulated hair follicle cells via peroxisome proliferator-activated receptor, which has been detected in three isoforms (PPAR*α*, PPAR*γ*, and PPAR*δ*) [44]. In contrast, mature adipocytes have a negative effect on the proliferation of hair follicles, as well as the proliferation of fibroblasts surrounding the follicle in the cocultures [18, 48].

Interestingly, changing the properties of the adipocyte cell lines may cause skin and hair disorders. Lipid metabolism may lead to defects in the structure of the skin and its functions. Overexpression of human apolipoprotein C1 (APOC1) with hyperlipidemia in transgenic mice results in abnormal hair growth correlated with the expression of the human APOC1 gene in the skin [18, 49].

Musina et al. assessed the influence of hypoxia as a stimulating factor for ADSCs to secrete growth factors. Subcutaneous injection induces the anagen phase in mice, as well as increases the proliferation of human follicular cells, keratinocytes, and hair papillae. Under the influence of hypoxia, there is an increased secretion of insulin-like growth factor binding protein- (IGFBP-) 1 and 2, M-CSF, M-CSF receptor, PDGF-*β*, VEGF, and decreased EGF secretion [12].

Unfortunately, the studies proved that the two-dimensional (2D) culture of the papilla cells lose their ability to form the hair (trichogenicity) and require a spheroidal form (3D) in culture [50, 51], Table 1.

## 5. Chronic Wounds

Damage to the skin leads to debilitating effects forming wounds. A wound is defined as a disruption of the normal anatomic structure and functional integrity of the skin. Chronic or nonhealing wounds are wounds that do not progress through the normal wound healing process, resulting in an open laceration of varying degrees of severity [9, 52]. Impaired healing is often associated with ischemia, diabetes mellitus, tumor, venous and pressure ulcers, and severe infections, and it can be the cause of reduced quality of life, disability, and even death [9, 53]. Therefore, wound healing remains a major challenge, and there is a need to develop treatments for improved therapy. Among the various strategies, the most promising seems to be the use of stem cells. This process remains a challenge to date and causes debilitating effects with tremendous suffering. Recent advances in tissue engineering approaches in the area of cell therapy have provided promising treatment options to meet the challenges of impaired skin wound healing [9].

Wound healing is a complex process, covering four mutually overlapping phases: hemostasis, inflammation, proliferation, and remodeling [5, 54, 55]. For the proper process to proceed, all steps must occur in the correct order and time [9]. In many chronic wounds, the elongation inflammatory phase leads to the damage of normal tissues, the production of an excessive amount of proinflammatory cytokines, and the prolonged presence of neutrophils, which causes the degradation of the extracellular matrix (ECM) due to an increase in the secretion of matrix metalloproteinases (MMPs) [9, 56].

Restoring the integrity of the skin involves several cell types, extracellular matrix components, and cytokines [57]. It is believed that what is physiologically responsible for the renewal of epidermal stem cells is located only in the basal layer of the epidermis. However, after damage to the skin, stem cells “bulge” in the region of the hair follicle and take additional responsibility for skin regeneration, particularly in the initial stage [4, 58].

Cell cultures enriched with stem and progenitor cells can be administered to patients via various methods: a direct application on the wound (e.g., as a suspension), injectable (arteriography), intravenous administration, or application of the culture on the appropriate biological scaffold. The most populous cells are the autologous progenitor cells of the epidermis. Current research is focused on bone marrow and adipose-derived stem cells being used in wound healing [4, 58].

ADSCs are involved in the process of healing indirectly by secreting a number of growth factors (IGF, TGF-*β*1, VEGF, HGF, and FGF2) with a paracrine action that activates keratinocytes and fibroblasts of the skin by stimulating the processes of neovascularization through the generation of anti-inflammatory cytokines, as well as having antioxidant and antiapoptotic effects [14, 36, 59, 60]. ADSCs release wound healing factors and can stimulate recruitment, migration, and proliferation of endogenous cells in the wound environment. The studies suggest that ASCs can affect other cell types specifically in skin tissue via the paracrine method [9]. They may also be directly transformed into fibroblasts and keratinocytes.

Human ADSCs can be converted to epithelial cells expressing the characteristics of cytokeratins 5, 14, 19, and *α*6; integrins; and even desmoglein 3 [60, 61]. They can also differentiate into fibroblast cells, demonstrating not only their morphological similarity but also their ability to also express cell surface proteins including vimentin and fibronectin [60]. An important feature of the ADSCs is that they produce an antioxidant that protects fibroblasts from oxidative stress [36]. One of the factors that induce ADSCs to increase secretion of growth factors and antiapoptotic factors is hypoxia at the wound site. It has been shown that culturing the ADSCs under hypoxic conditions improves their ability to bind to the adhesion molecules (ICAM-1, VCAM-1), which leads to faster neovascularization, increased production of bFGF, and increased ADSC proliferation [9, 16, 36, 62]. A recent study provides evidence that stromal cell-derived factor-1 (SDF-1) can increase the therapeutic effect of ADSCs in cutaneous chronic wounds. It may protect against cell apoptosis in hypoxic and serum-free conditions through activation of the caspase signaling pathway in ADSCs [63].

The first attempts at healing chronic wounds were performed using ADSCs from lipoaspirate, even without culturing in vitro [9]. This technique is commonly used in aesthetic medicine, avoiding the manipulation that might influence their biological functioning. The simplest method is the application of a component of the adipose tissue-derived multicellular stromal vascular fraction (SVF), after enzymatic digestion and centrifugation of lipoaspirate [64, 65]. SVF is a heterogeneous population of MNCs that include ADSCs of the mesenchymal phenotypes (analogous to MSCs), endothelial progenitor cells (EPCs), hemopoietic progenitors, monocytes, leukocytes, and pericytes. Pericytes are the most important for angiogenesis, and they stabilize nascent blood vessels [65–67].

The administration of wound single-cell suspensions often leads to the formation of aggregates and islet necrosis which can occur after cell injection. Monolayer-cultured cells are poorly retained in local transplantations, nullifying the therapeutic intent or resulting in unexpected stem cell behaviors [68, 69]. Then, the low cell engraftment efficiency by injection approach has significantly limited the clinical translation of stem cell therapies [70]. Some techniques use matrices like atelocollagen or the scaffolding of silk fibroin-chitosan suspension ADSCs [71, 72]. Sivan et al. used fibrin and fibronectin to construct an in vitro niche and the mimicking of an in vivo provisional matrix, which plays a dual role in the support of hemostasis, accelerates cell attachment and growth, and is responsible for the increased survival of differentiated cells [73].

Nowadays, researchers are focused on the three-dimensional (3D) culture systems of ADSCs to build multicellular constructs with an extracellular matrix (ECM) and to demonstrate better therapeutic efficacy [68, 69]. The study by Cerqueira et al. used human ADSCs with an extracellular matrix (ECM) as a natural tissue glue that was applied to three layers to form a 3D structure (these are known as “technique sheets”). Then, they were transferred to wounds in mice, obtained by the complete excision of the skin. Restoration of the skin was observed with the formation of new hair follicles and vessels. This resulted in a greater stability of transplanted ADSCs, through cell-cell and cell-ECM interactions. The sheet technique greatly improves the efficiency of transplanted ADSCs [14]. Feng et al. describe a simple method for the 3D culture of adipose-derived stem/stromal cells (ADSCs) which prepares them into a ready-to-use injectable. They transferred suspensions of monolayer-cultured ASCs to a syringe containing hyaluronic acid gel (a naturally derived ECM component) and then incubated the syringe as a 3D culture vessel (microspheroids of human ADSCs). They confirmed high therapeutic efficacy in pathological wound repair in vivo [68]. However, central necrosis was reported when spheroids of mesenchymal stem cells reached a diameter of 200 *μ*m in a suspension-rocking culture system [63, 68]. There are high hopes for a new technology that uses a semi-interpenetrated polymer network (semi-IPN) structure. It was developed by combining this polymer with hyaluronic acid (HA), leading to an in situ cross-linkable hydrogel with significantly increased porosity, enhanced swelling behavior, and improved cell adhesion and viability in both 2D and 3D cell culture models [74].

The next step in the current research is looking for additional materials that may resemble a physiological niche for stem cells to enhance cell retention. Conditioned media for ADSCs have been reported to enhance angiogenesis, enhance epithelialization, and affect recruitment or proliferation of macrophages and endothelial progenitor cells during the healing process [75]. Dong et al. have developed a method of using an injectable poly(ethylene glycol) (PEG)-gelatin hydrogel with highly tunable properties. Murine ADSCs can be easily encapsulated into the hydrogel, which supports ADSC growth and maintains their stemness. This method significantly improves cell retention, enhances angiogenesis, and accelerates wound closure using a murine wound healing model. Then, the injectable PEG-gelatin hydrogel can be used for regulating stem cell behaviors in 3D culture or to deliver cells for wound healing and other tissue regeneration applications [70]. It was found that long-term cell viability could be achieved for both in vitro (21 days) and in vivo (14 days) studies. With ADSCs, this hydrogel system showed potential as a bioactive hydrogel dressing for wound healing [75].

On the basis of many studies, the best wound healing is achieved by using ADSCs with platelet-rich plasma (PRP). Their presence has caused more rapid proliferation of fibroblasts and keratinocytes in vitro [57, 76–78]. PRP is a source of growth factors, necessary for healing, such as PDGF, TGF, IGF, and EGF. They are concentrated in the platelets. In addition, PRP can act as a scaffold for other types of cells such as mesenchymal stem cells [57, 76, 79]. On the other hand, higher concentrations of PRP in vitro culture can slow down the rate of regeneration due to proteolytic enzymes (PRP-collagenase, elastase, and cathepsin) which inhibit cell growth. The best results have been achieved after using a maximum 10% PRP [57, 78].

Healing of chronic cutaneous wounds and ulcers is troublesome and may require the use of skin substitutes. Adipose-derived stem cells have immense potential as an autologous cell source for treating wounds and regenerating skin, Table 2.

## 6. Conclusions

ADSCs appear as the ideal cell population for the use in regenerative medicine:

- they are unlimited in supply and easily obtainable from adipose tissue;
- they are autologous, nonimmunogenic cells;
- they have a multipotential nature and easily differentiable into various cell lines;
- they have a significant potential of angiogenesis;
- they can be easily cultured and have high affinity for 3D scaffolds [2, 56, 60].

## Figures and Tables

**Table 1:** List of past and present clinical adipose-derived stem cell trials in alopecia [80].

Number	Study	Application method	Conditions	Phase	Trial institution/sponsor and country	NCT number and duration period
(1)	The Effect of Allogeneic Human Adipose Derived Stem Cell Component Extract on Androgenic Alopecia	Stem cell component extract	Androgenic alopecia	III	Pusan National University Hospital, South Korea	NCT02594046 2015-2016
(2)	Adipose Tissue Derived Stem Cell Based Hair Restoration Therapy for Androgenetic Alopecia (AGA)	Stem cells with platelet-rich plasma	Androgenic alopecia	II	King Edward Medical University, Pakistan	NCT02865421 2016–2018
(3)	Biocellular-Cellular Regenerative Treatment Scaring Alopecia and Alopecia Areata (SAAA)	PRP concentration, emulsification tSVF	Alopecia areata Scarring alopecia		Regeneris Medical, USA	NCT03078686 2017–2019
(4)	AGA Biocellular Stem/Stromal Hair Regenerative Study (STRAAND)	Emulsified adipose-derived tissue stromal vascular fraction with platelet-rich plasma	Hair disease	I, II	Healeon Medical Inc./Ministry of Health, Honduras	NCT02849470 2016–2018

**Table 2:** List of past and present clinical adipose-derived stem cell trials on chronic wound [80].

Number	Study	Application method	Conditions	Phase	Trial institution/sponsor and country	NCT number and duration period
(1)	Adipose derived Regenerative Cellular Therapy of Chronic Wounds	Injections to the wound	Venous ulcer, diabetic foot, pressure ulcer	II	Tower Outpatient Clinic, Los Angeles, California, USA	NCT02092870 2013–2015
(2)	ADSCs and Pressure Ulcers	Injections (stromal vascular fraction) to rim around the venous stasis of the wound	Chronic venous stasis wounds	I	Sanford Plastic and Reconstructive Surgery Clinic, Sioux Falls, USA	NCT02961699 2017–2020
(3)	ADSCs and Pressure Ulcers	Injected into a fibrin sealant applicator and applied to the wound	Pressure ulcer	I	Mayo Clinic, Florida, USA	NCT02375802 2015–2017
(4)	Safety of Adipose-Derived Stem Cell Stromal Vascular Fraction	Injections with or without unprocessed autologous fat (fresh or cryopreserved)	Abnormally healing wounds, scars, and soft tissue defects	I	AdiSave Inc., Canada	NCT02590042 2015–2019
(5)	Assessment of the Efficacy and Tolerance of Sub-cutaneous Re-injection of Autologous Adipose-derived REGEnerative Cellsin the Local Treatment of Neuropathic Diabetic Foot ulcERs (REGENDER)	Injections to the wound	Diabetic foot ulcer	II	Assistance Publique Hopitaux De Marseille, France	NCT02866565 2017–2019
(6)	Treatment of Hypertensive Leg Ulcer by Adipose Tissue Grafting	Adipose tissue grafting (lipofilling)	Hypertensive leg ulcer	I	University Hospital, Caen, France	NCT01932021 2013–2014
(7)	Safety of ALLO-ASC-DFU in the Patients with Diabetic Foot Ulcers	ALLO-ASC-DFU	Diabetic foot ulcer	I	Asan Medical Center, Seoul, South Korea	NCT02394886 2014–2015
(8)	The Role of Lipoaspirate Injection in the Treatment of Diabetic Lower Extremity Wounds and Venous Stasis Ulcers	Injection of lipoaspirate	Diabetic wounds, venous stasis wounds	—	Washington DC Veterans Affairs Medical Center, USA	NCT00815217 2009–2010
(9)	Allogeneic ADSCs and Platelet-Poor Plasma Fibrin Hydrogel to Treat the Patients With Burn Wounds	Applied by surface application over perforated (1 : 3) autologous skin graft	Second- or third-degree burns	I, II	The Kyiv City Clinical Hospital, Ukraine	NCT03113747 2015–2018
(10)	Safety of ADSCs Stromal Vascular Fraction	Injections (stromal vascular fraction) to the wound fresh or cryopreserved autologous fat	Abnormally healing wounds, scars, and soft tissue defects	I	Forest Hill Institute of Aesthetic Plastic Surgery, Toronto, Ontario, Canada	NCT02590042 2015–2019
(11)	Child's Adipose Cells: Capacity of Tissue Regeneration	Adipose tissue sample in children < 10 y.	A nude mouse model of skin burn	—	CHU de Toulouse-Purpan, Tolouse, France	NCT02779205 2015–2017
(12)	A Study to Evaluate the Safety of ALLO-ASC-DFU in the Subjects With Deep Second-degree Burn Wound	A hydrogel sheet containing allogenic ASC	Deep second-degree burn wound patients	I	Hallym University of Medical Center, Seoul, Republic of Korea	NCT02394873 2015
(13)	ADSCs for the Treatment of Perianal Fistula in Crohn Disease: A Pilot Study (ASPEFIC1)	ADSC injection	Perianal fistula Crohn's disease	II	Papa Giovanni XXIII Hospital, Italy	NCT02403232 2014–2018
(14)	Autologous Cultured Adipose-Derived Stem Cells (ANTG-ASC) on Complex and Crohn's Fistula	ADSC injection	Perianal fistula Crohn's disease	II	Seoul National University Hospital, Republic of Korea	NCT01314092 2011–2016
(15)	Adipose-Derived Stem Cells to Treat Complex Perianal Fistulas	ADSC injection and dressing with fibrin glue	Perianal fistulas	I	General Surgery Department, Hospital Universitario La Paz, Spain	NCT01020825 2009–2011
